# Cardiovascular Disease and Stroke Risk Among Egyptian Resident Physicians: A Cross-Sectional Multicenter Study

**DOI:** 10.7759/cureus.58024

**Published:** 2024-04-11

**Authors:** Alaa Ramadan, Mostafa A Soliman, Abdullah A Hamad, Mohamed El-Samahy, Merna R Roshdy, Rehab A Diab, Yomna E Abdalla, Moamen Emara, Asmaa K Azooz, Dina S Abo El-lail, Eman H Elbanna, Mohammed E Almalki, Basel Abdelazeem, Ahlam S Ali, Ahmed Negida

**Affiliations:** 1 Department of Medicine, South Valley University, Qena, EGY; 2 Department of Medicine, Cairo University, Cairo, EGY; 3 Department of Medicine, Menoufia University, Shebin El-Koum, EGY; 4 Department of Medicine, Zagazig University, Zagazig, EGY; 5 Department of Medicine, Sohag University, Sohag, EGY; 6 Department of Medicine, Al-Azhar University, Cairo, EGY; 7 Department of Medicine, Port Said University, Port Said, EGY; 8 Department of Pharmacy, Helwan University, Helwan, EGY; 9 Department of Health Administration and Behavioral Sciences, High Institute of Public Health, Alexandria University, Alexandria, EGY; 10 Department of Medicine, Umm Alqura University, Makkah, SAU; 11 Department of Internal Medicine, McLaren Health Care, Flint, USA; 12 Department of Internal Medicine, Michigan State University, East Lansing, USA

**Keywords:** health policy, egypt, qrisk3, physicians, cardiovascular disease

## Abstract

Background: Cardiovascular diseases (CVDs) are the leading cause of death worldwide and are considered silent killers that threaten different age groups. The stressful lifestyle of resident physicians might make them vulnerable to CVDs. Since 2021, Egypt has recently reported more frequent sudden deaths of junior physicians after long shifts. Many factors can be associated with this prevalence, such as diabetes mellitus, increased blood pressure, or a sedentary lifestyle. Therefore, this study aimed to estimate the risk of developing heart attack and stroke within 10 years among resident physicians in Egypt with the goal of informing health policymakers to improve the healthcare systems for Egyptian physicians.

Methods: This cross-sectional study was conducted at six university teaching hospitals around Egypt: Cairo, Al-Azhar, Zagazig, Menoufia, South Valley, and Sohag. Data were collected on the ground using a questionnaire developed from a validated tool, the QRISK3 calculator, developed by the National Health Service, and used to measure the development of CVDs and stroke over the next 10 years.

Results: Four hundred twenty-eight resident physicians filled out the study questionnaire, including 224 (52.3%) females. The mean age of the participants was 28.22 years (±2.54). The study revealed that 258 (60.3%), with a median (IQR) = 0.2% (0.1%-0.5%), of the resident physicians are at high risk of having a heart attack or stroke within 10 years. Migraine symptoms (n=65, 15.2%) and angina or heart attack in a first-degree relative (n=26, 6.1%) were the most reported risk factors. The risk was variable among the six university hospitals, with a significant P-value <0.001, where Menoufia University hospitals ranked first, followed by Zagazig University hospitals. However, the percentage of each specialty differs from others. The highest risk was among anesthesiology and ICU residents (n=18, 78.3%), followed by surgery residents (n=44, 62.9%).

Conclusion: About 258 (60.3%) of the resident physicians are at risk of having a heart attack or stroke within 10 years. There is an urgent need to increase resident physicians’ awareness about their heart attack and stroke risks and for health policymakers to ensure a better lifestyle and friendly training environment for resident physicians in Egypt.

## Introduction

Cardiovascular diseases (CVDs) are the leading causes of death worldwide, accounting for about 17.9 million deaths in 2019 [1]. Epidemiological studies identified several risk factors associated with CVDs as smoking tobacco, a sedentary lifestyle, elevated blood pressure, obesity, or diabetes mellitus. Other non-modifiable risk factors include age, gender, and family history [2]. The literature data showed that CVD risk factors are common in Arab countries' populations [3]. In Egypt, coronary heart disease deaths account for 32.40% of total deaths, making Egypt the 15th highest country with coronary heart disease deaths in the world, according to the latest WHO data [4]. The prevalence of CVD risk factors, management strategies, and health outcomes of CVD significantly vary according to geographical location [5]. Stroke, a critical subtype of CVD, holds a significant public health concern due to its high morbidity and mortality rates [6]. It occurs when the blood supply to the brain is disrupted, leading to the sudden loss of brain functions. The consequences of stroke can be devastating, often leading to long-term disability and impairments in movement, speech, and cognition [6]. Therefore, understanding the risk factors and incidence of stroke among specific populations, such as resident physicians in Egypt, is vital for informing health policymakers and optimizing healthcare systems to address this pressing issue.

Healthcare professionals (HCPs) are an important and diverse group of the population who spend most of their time on the diagnosis and management of health-related conditions. It is reasonable to think they lead healthy lifestyles and have better health than others [7]. There is a strong relationship between the health and behavior of HCPs and the provision of health care or good counseling to their patients. This is expressed in the extent to which HCPs adhere to healthy habits that benefit their physical health [8]. Many studies indicate the risk of CVD among healthcare workers; according to a study in Malaysia, 42% of healthcare workers had at least one medical problem, such as dyslipidemia or diabetes mellitus [9]. However, some studies indicated that most HCPs do not have the expected physical exercise levels and are significantly more vulnerable to CVDs [10]. The health status of HCPs is important to maintaining a strong healthcare system and should be a priority for health policymakers.

Resident physicians are an important part of the healthcare staff, either input by the patient care process or output by the training process [11]. Resident physicians have trouble admitting they are sick compared to the general population, which appears to be due to their fear of missing out on their training or speciality decisions [12]. On the other hand, time constraints appear to be an important factor in the neglect of their illness due to their constant preoccupation [13]. In addition, uncooperative seniors or an unfriendly work environment might create a stressful lifestyle for resident physicians. Since 2021, Egypt has witnessed an unprecedentedly high rate of sudden deaths among young physicians. Therefore, this study aimed to identify the risk of developing a heart attack or stroke within 10 years among resident physicians in Egypt to inform health policymakers and optimize the healthcare systems for Egyptian physicians.

## Materials and methods

We conducted a cross-sectional study within the period from April 20, 2022 to May 15, 2022, in six university teaching hospitals in Egypt: Cairo University Hospital (Kasr Alainy), Al-Azhar University Hospital (Al Zahraa Hospital), Zagazig University Hospital (Sednawi Hospital), Menoufia University Hospital (Shebin El-Kom Hospital), South Valley University Hospital (Marzouqi Hospital), and Sohag University Hospital. We used a survey-based method to collect data for this study. Eligible participants in the self-selected university hospitals were asked to fill out the survey. The main aim of this study was to assess the prevalence of risk factors for stroke and heart attack among resident physicians in Egypt. Although cross-sectional studies are not typically designed to predict the risk of developing these conditions over a long period, they provide valuable insights into the prevalence and distribution of risk factors within a specific population at a given time. However, certain scientists have developed algorithms, such as the QRISK3 calculator, aimed at predicting the 10-year risk of heart attack and stroke based on current risk factors. These algorithms estimate the relative risk (RR) in comparison to matched controls of the same age, sex, and ethnicity. This study was approved by the Ethics Committee of South Valley University (approval number: 4/394). The confidentiality and anonymity of the participants were explained, and informed consent was obtained from the participants before filling out the questionnaires. We followed the Strengthening the Reporting of Observational Studies in Epidemiology statement guidelines when reporting this manuscript (Table 4 in Appendix) [14].

Data collection

The target population of this study is defined as resident physicians who are currently in the residency training years in Egyptian Universities' hospitals. Within this large target population, we self-selected six university hospitals that were accessible to the research team. The sample population was resident physicians in all the clinical departments of the six university hospitals of both sexes and aged >25 years. We excluded undergraduates, intern students, and nursing staff. The total sample size required for this study was calculated based on sample size calculation methods for a single proportion [15] using an online sample size calculator [16]. Assuming a CVD risk of 49.2%, as reported in a previous study by Hegde et al. [17], and a population size of 1224 according to the Egyptian Ministry of Higher Education and Scientific Research statistics [18], a minimum sample size of 410 participants was required to detect a similar proportion rate with 95% confidence intervals and 5% precision. This sample size was distributed among the six university hospitals around Egypt. We collected the data initially using an online survey, but for some locations with limited internet access, we provided hard copies of the questionnaire. Participants were asked to submit the online questionnaires on smartphones or fill in the hard copies of the questionnaires. Irrespective of the data collection method, all responses were deposited in a single database and coded for analysis. To assess the prevalence of risk factors for stroke and heart attack among resident physicians in Egypt and predict the risk of developing these conditions, we utilized the QRISK3 calculator [19]. Developed by the National Health Service in the UK, this online tool incorporates 22 risk factors to calculate the 10-year risk of heart attack and stroke, while also estimating the RR compared to matched controls of the same age, sex, and ethnicity. The QRISK3 calculator provided a comprehensive assessment of various risk factors, including age, sex, ethnicity, smoking status, diabetes status, history of angina or heart attack in a first-degree relative under the age of 60, chronic kidney disease (stage 3, 4, or 5), atrial fibrillation, blood pressure treatment, migraines, rheumatoid arthritis, systemic lupus erythematosus, severe mental illness (including schizophrenia, bipolar disorder, and moderate/severe depression), atypical antipsychotic medication, regular steroid tablets, diagnosis or treatment for erectile dysfunction, cholesterol/high-density lipoprotein ratio, systolic blood pressure, the standard deviation of at least two most recent systolic blood pressure readings, height, and weight. In this study, individuals with a risk of developing heart attack and stroke over the 10-year period were defined as those with an RR greater than one in the QRISK score. By utilizing the QRISK3 calculator, we aimed to identify individuals at higher risk and provide insights into the prevalence and distribution of risk factors associated with these conditions among resident physicians in Egypt. However, it is important to note that the QRISK3 calculator provides risk estimations and should be interpreted as such, with further longitudinal studies needed to validate and refine these predictions.

Statistical analysis

Qualitative data were presented as frequencies and percentages, and quantitative variables were presented as mean and standard deviation. Participants were divided into two groups based on their RR of developing a heart attack or stroke over the next 10 years: no risk factor group included participants having an RR of less than or equal to one, and the risk factor group included those having an RR of greater than one. A comparison of different specialties and hospitals by the RR of developing a heart attack or stroke over the next 10 years was made using the chi-square test. A P-value of 0.05 or less is considered statistically significant. We used IBM SPSS Statistics for Windows, Version 28 (Released 2021; IBM Corp., Armonk, New York, United States) to analyze the data.

## Results

Four hundred twenty-eight resident physicians filled out the study questionnaire, including 204 (47.7%) males and 224 (52.3%) females. The mean age of the participants was 28.22 years (±2.54), with a BMI of 26.56 kg/m^2^ (±3.66) and mean systolic blood pressure of 118.81 mmHg (±9.91). Table 1 shows a summary of the sociodemographic data for all participants.

**Table 1:** The sociodemographic characteristics of the study population The data is presented in the form of mean ±SD or n(%). BMI: Body mass index

Variables		Frequency (percentage)
Age (mean±SD)		28.22 (2.54)
Sex		-
Male		204 (47.7%)
Female		224 (52.3%)
BMI (mean±SD)		26.56 (3.66)
Systolic blood pressure (mmHg)		118.81 (9.91)
Marital Status		-
Single		213 (49.8%)
Engaged		59 (13.8%)
Married		155 (36.2%)
Divorced		1 (0.2%)

Out of 428 participants, 258 (60.3%) of them had a risk of developing a heart attack or stroke over the next 10 years, with median (IQR) = 0.2% (0.1%-0.5%). The median of RR among them was 1.1 with IQR (0.8-1.5). The most reported risk factor was 65 (15.2%) migraines following 26 (6.1%) a history of angina or a heart attack. All participants did not report a history of erectile dysfunction. Most participants have a BMI ranging from 25 kg/m^2^ to 30 kg/m^2^ with a mean of 26.56 kg/m^2^ (SD=3.67). Table 2 shows the prevalence of all CVD risk factors included in our study.

**Table 2:** The CVD risk factors in the studied population The data is presented in the form of n (%). SLE: Systemic lupus erythematosus; CVD: Cardiovascular disease

Variables	Frequency (percentage)
Smoking status	-
Non-smoker	411 (96%)
Ex-Smoker	5 (1.2%)
Light smoker (less than 10/day)	8 (1.9%)
Moderate smoker (10 to 19 cigars/day)	3 (0.7%)
Heavy smoker (over 20 cigars/day)	1 (0.2%)
Diabetic status	
Non-diabetic	424 (99.1%)
Type II diabetes mellitus	4 (0.9%)
Angina or heart attack in a first-degree relative < 60?	
Yes	26 (6.1%)
No	402 (93.9%)
Chronic kidney disease (stage 3, 4, or 5)?	
Yes	3 (0.7%)
No	425 (99.3%)
Atrial fibrillation?	
Yes	3 (0.7%)
No	425 (99.3%)
On blood pressure treatment?	
Yes	23 (5.4%)
No	405 (94.6%)
Do you have migraines?	
Yes	65 (15.2%)
No	363 (84.8%)
Rheumatoid arthritis?	
Yes	3 (0.7%)
No	425 (99.3%)
SLE?	
Yes	1 (0.2%)
No	427 (99.8%)
Severe mental illness? (this includes schizophrenia, bipolar disorder, and moderate/severe depression)	
Yes	4 (0.9%)
No	424 (99.1%)
On atypical antipsychotic medication?	
Yes	1 (0.2%)
No	427 (99.8%)
Are you on regular steroid tablets?	
Yes	4 (0.9%)
No	424 (99.1%)
A diagnosis of or treatment for erectile dysfunction?	
No	428 (100%)

The prevalence of CVD risk factors was variable between physicians from six university hospitals (P<0.001). About 90.5% of Menoufia Hospital's physicians are at risk of developing CVD in the next 10 years, while 73.8% of Zagazig University Hospital's physicians are at risk of developing CVD in the next 10 years. Table 3 presents the difference in the prevalence of risk factors for CVDs between physicians from the six university hospitals.

**Table 3:** Sociodemographic data characteristics The data is presented in the form of n(%). RR: Relative risk

Hospital	Not at risk Q-risk RR≤1 n=170 (39.7%)	At risk Q-risk RR>1 n=258 (60.3%)	Total n=428	P-value
Cairo University Hospitals	35 (44.9%)	43 (55.1%)	78 (18.2%)	<0.001
Zagazig University Hospitals	32 (26.2%)	90 (73.8%)	122 (28.5%)	
Menoufia University Hospitals	2 (9.5%)	19 (90.5%)	21 (4.9%)	
Al-Azhar University Hospitals	42 (54.5%)	35 (45.5%)	77 (18%)	
South Valley University Hospitals	26 (49.1%)	27 (50.9%)	53 (12.4%)	
Sohag University Hospitals	33 (42.9%)	44 (57.1%)	77 (18%)	

**Figure 1:**
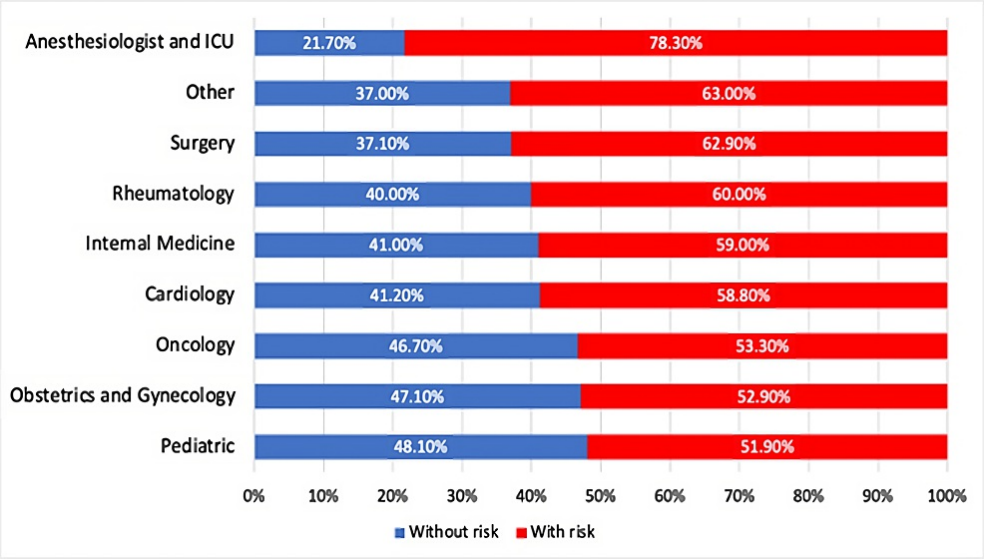
Comparison of different specialties by the relative risk of developing a heart attack or stroke over the next 10 years

No significant difference was found in the risk of developing a heart attack or stroke over the next 10 years among physicians from different specialties (P=0.6). Anesthesiology and ICU residents had the highest risk (78.3%) of developing a heart attack or stroke over the next 10 years, while surgery residents ranked second. Figure 1 shows the prevalence of the risk factors of CVDs among all specialties.

## Discussion

This study revealed that out of the 428 studied participants, 258 (60.3%) were at high risk of developing heart attacks and strokes over the upcoming 10 years. In terms of the risk factors, migraine symptoms (15.2%) and angina or heart attack in a first-degree relative <60 (6.1%) were the most reported risk factors.

There was a difference in cardiovascular risk among resident physicians from different specialties. Anesthesiologists and ICU residents were the highest risk groups, with a risk of about 78.3%, followed by surgeons (62.9%). This result is expected due to a large number of working hours associated with psychological stress among physicians of these specialties, which results in a complete lack of awareness of their physical health and an increase in their incidence of CVDs [20]. Almost half of the resident physicians were single, associated with an increased risk of CVDs. Marriage can be a protective factor against many risks that increase the possibility of developing diseases of the body in general and CVDs in particular. This is represented by the interest of both parties in the health of each other and in providing psychological and social support [21]. Our results showed the prevalence of risk factors for CVDs was variable among physicians from six university hospitals, with a significant P-value <0.001. Menoufia ranked first with 90.5%, followed by Zagazig with 73.8%. These results agreed with Hassanin's study in 2020, which showed an association between the demographic and clinical characteristics of hospitalized heart failure patients in different regions of Egypt because of the different prevention and management strategies around Egypt [5]. About 318 (74.2%) of the participants didn't measure their cholesterol/high-density lipoprotein ratio, indicating the absence of continuous follow-up. This aligns with other studies that revealed unawareness of health behaviors and risks among healthcare workers [22]. The present data demonstrated high BMI among our study sample of resident physicians; BMI (26.56±3.67) indicated that most participants were classified as overweight, which indicates a higher risk of developing CVDs [23]. This finding is largely concordant with the study done in the USA by the Harvard School of Public Health, which found that about 64% of the healthcare workers included in this study were overweight with a BMI ≥25 kg/m^2^ [7]. Our current study also revealed that about 15% of resident physicians take migraine medications. The appearance of migraine symptoms and taking their medications are associated with an increased risk of ischemic stroke and CVDs [24]. High blood pressure is one of the most important risk factors for CVD, associated with high morbidity and mortality rates [25]. The relationship between systolic blood pressure (118.8±9.91 mmHg) and the RR for CVDs is presented in our study among 362 resident physicians, which reveals the marked risk of developing CVDs among them. Evaluating the risk of developing a heart attack or stroke over the next 10 years is an important indicator of avoiding many expected health problems. The selection of healthcare workers, in particular, represents a solid basis for the general population's health.

Limitations

Some limitations of the study should be mentioned. This study is cross-sectional, so it does not establish a temporal relationship between the CVD risk factors and the residency training or specialty choice. Further prospective studies are recommended to identify the risk factors of CVDs in HCPs compared to the general population and to examine the impact of lifestyle modifications on CVDs risk among physicians. This information warrants health policymakers to improve the current working environments for junior physicians in Egypt.

## Conclusions

In conclusion, our study reveals a high prevalence of risk factors for stroke and heart attack among Egyptian resident physicians. Approximately 60% of the participants were found to be at risk of developing these cardiovascular conditions within the next 10 years, as indicated by the QRISK3 calculator. These findings emphasize the urgent need to enhance awareness among resident physicians regarding their individual risks for heart attack and stroke. Furthermore, it highlights the importance for health policymakers to prioritize the implementation of measures that promote a healthier lifestyle and create a supportive training environment for resident physicians in Egypt.

## Appendices

**Table 4:** STROBE statement - checklist of items that should be included in reports of cross-sectional studies STROBE: Strengthening the Reporting of Observational Studies in Epidemiology

Item name	Item no	Recommendation	
Title and abstract	1	(a) Indicate the study’s design with a commonly used term in the title or the abstract	1
		(b) Provide in the abstract an informative and balanced summary of what was done and what was found	2
Introduction			
Background/rationale	2	Explain the scientific background and rationale for the investigation being reported	4
Objectives	3	State-specific objectives, including any prespecified hypotheses	5
Methods			
Study design	4	Present key elements of study design early in the paper	6
Setting	5	Describe the setting, locations, and relevant dates, including periods of recruitment, exposure, follow-up, and data collection	6
Participants	6	(a) Give the eligibility criteria and the sources and methods of selection of participants	6
Variables	7	Clearly define all outcomes, exposures, predictors, potential confounders, and effect modifiers. Give diagnostic criteria, if applicable	7
Data sources/measurement	8	For each variable of interest, give sources of data and details of methods of assessment (measurement). Describe comparability of assessment methods if there is more than one group	8
Bias	9	Describe any efforts to address potential sources of bias	NR
Study size	10	Explain how the study size was arrived at	6
Quantitative variables	11	Explain how quantitative variables were handled in the analyses. If applicable, describe which groupings were chosen and why	8
Statistical methods	12	(a) Describe all statistical methods, including those used to control for confounding	8
		(b) Describe any methods used to examine subgroups and interactions	NA
		(c) Explain how missing data were addressed	NA
		(d) If applicable, describe analytical methods taking account of sampling strategy	NA
		(e) Describe any sensitivity analyses	NA
Results			
Participants	13	(a) Report numbers of individuals at each stage of study - eg numbers potentially eligible, examined for eligibility, confirmed eligible, included in the study, completing follow-up, and analyzed	9
		(b) Give reasons for non-participation at each stage	NA
		(c) Consider the use of a flow diagram	NA
Descriptive data	14	(a) Give characteristics of study participants (eg demographic, clinical, social) and information on exposures and potential confounders	9-10
		(b) Indicate the number of participants with missing data for each variable of interest	9-10
Outcome data	15	Report numbers of outcome events or summary measures	9-10
Main results	16	(a) Give unadjusted estimates and, if applicable, confounder-adjusted estimates and their precision (eg, 95% confidence interval). Make clear which confounders were adjusted for and why they were included	NA
		(b) Report category boundaries when continuous variables were categorized	NA
		(c) If relevant, consider translating estimates of relative risk into absolute risk for a meaningful time period	NA
Other analyses	17	Report other analyses are done - eg analyses of subgroups and interactions, and sensitivity analyses	NA
Discussion			
Key results	18	Summarise key results with reference to study objectives	11
Limitations	19	Discuss limitations of the study, taking into account sources of potential bias or imprecision. Discuss both the direction and magnitude of any potential bias	12
Interpretation	20	Give a cautious overall interpretation of results considering objectives, limitations, multiplicity of analyses, results from similar studies, and other relevant evidence	11-12
Generalisability	21	Discuss the generalisability (external validity) of the study results	12
Other information			
Funding	22	Give the source of funding and the role of the funders for the present study and, if applicable, for the original study on which the present article is based	13
